# 5-(Pyridin-3-yl)-3,4-dihydro-2*H*-furan-1-ium (NNKFI): a computational study of its physico-chemical properties

**DOI:** 10.1098/rsos.230975

**Published:** 2024-09-11

**Authors:** Evan Millam, Christos Deligkaris, Edmir O. Wade

**Affiliations:** ^1^ Department of Chemistry and Biochemistry, University of Southern Indiana, Evansville, IN 47712, USA; ^2^ Department of Geology and Physics, University of Southern Indiana, Evansville, IN 47712, USA

**Keywords:** 5-(pyridin-3-yl)-3,4-dihydro-*2H*-furan-1-ium, computational, oxonium, furanium, properties, alkylation

## Abstract

Recent work on the diazonium ion metabolite of 4-(methylnitrosamino)-1-(3-pyridyl)-1-butanone (NNKDI) suggests that 5-(pyridin-3-yl)-3,4-dihydro-2*H*-furan-1-ium (NNKFI) may form from NNKDI via an intramolecular reaction. NNKDI is an important carcinogen whose role as an alkylating agent has received significant attention. While there is some experimental evidence supporting NNKFI’s production *in vitro*, it has not yet been directly observed. Little is known about NNKFI’s structure and reactivity. We report the first *in silico* examination of this ion. Our study utilized Kohn–Sham density functional theory (B3LYP/6-311G**) and coupled cluster theory (CCSD/6-31G*) to produce energy-optimized structures, vibrational normal modes and molecular orbitals for NNKFI. To gain insight into the chemical properties of this species, we calculated electrostatic potential surfaces, natural population analysis charges and local Fukui indices. We report data and results for NNKFI’s *cis* and *trans* conformers. Our work confirms C5 as the preferred site for nucleophilic attack in NNKFI. Stretching motions and predicted bond lengths near O1 are consistent with a somewhat weakened carbonyl structure in this ion. Partial charges, electrostatic potential surfaces and local Fukui indices reveal delocalization of cationic charge on the furanium moiety and notable carbocation character at C5.

## 1. Introduction

**Figure 1.**
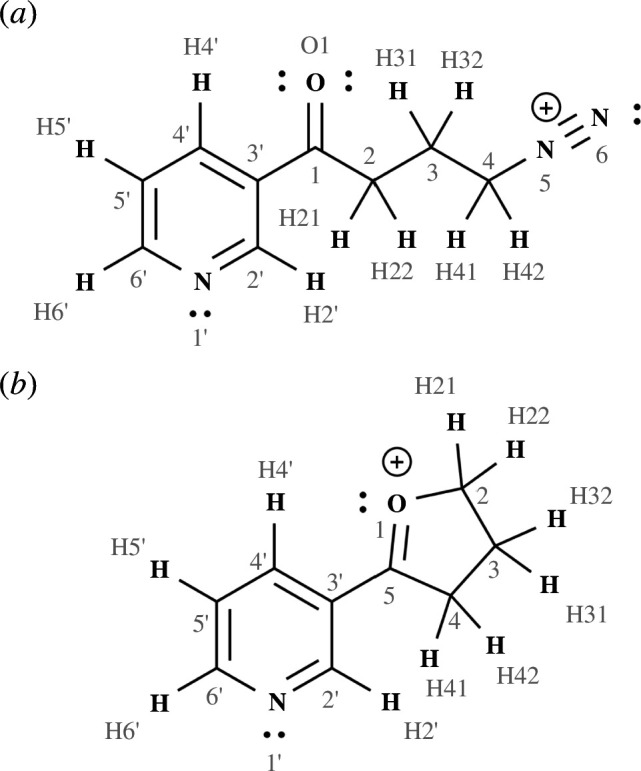
4-(Methylnitrosamino)-1-(3-pyridyl)-1-butanone diazonium ion metabolite (NNKDI) (*a*) and 5-(pyridin-3-yl)-3,4-dihydro-2*H*-furan-1-ium (NNKFI) (*b*).

The International Agency for Research on Cancer (IARC) classifies 4-(methylnitrosamino)-1-(3-pyridyl)-1-butanone (NNK) as ‘carcinogenic to humans’ (Group 1) [1]. NNK is a tobacco-specific nitrosamine found in relatively high concentrations in mainstream cigarette smoke [2–6]. Its diazonium ion metabolite, NNKDI (figure 1), acts as an alkylating agent [5–9] and is responsible for pyridyloxobutyl (POB) adduct formation [5–14], a part of carcinogenesis [15–18]. When exposed to neutral or acidic conditions, many POB adducts undergo thermal hydrolysis producing 4-hydroxy-1-(3-pyridyl)-1-butanone (HPB) [5,7,11,15,17,19–29]. While POB adducts themselves have not yet been observed in humans [30], HPB-releasing adducts have been found and are considered a biomarker of NNK exposure [5,27,31–37].

Our recent work [38] on NNKDI suggested that 5-(pyridin-3-yl)-3,4-dihydro-2*H*-furan-1-ium (NNKFI) (figure 1) forms spontaneously during internal rotation along the diazonium ion’s butanone side chain. These calculations examined NNKDI’s rotational potential energy surface and confirmed both the nucleophilicity of NNKDI O1 and the electrophilicity of NNKDI N5 and N6. Partial charge predictions also implied an electrostatic attraction between NNKDI N6 and O1. Conformers that positioned NNKDI C4 proximate to O1 led to dinitrogen loss and furanium ion formation during energy minimization.

This intramolecular reaction has been studied previously. Solvolysis of various diazonium ion and furanium ion precursors in methanol implied the production of NNKFI from NNKDI. In the same study, ring opening was seen when NNKFI reacted with hydroxide (re-forming the carbonyl at NNKFI C5, breaking the O1–C2 bond, and attaching the substrate at NNKFI C2) [39]. Thus, ring opening may make NNKFI and NNKDI alkylation products difficult to distinguish. An analogous class of six-membered cyclic oxonium ions, glycosyl oxocarbenium ions, have been observed experimentally and are thought to be important intermediates in glycosylation [40].

Upon reaction with DNA, NNKFI is expected to produce 2-(3-pyridyl)-2,3,4,5-tetrahydrofuan (PTF) adducts [5,17]. PTF adducts have not been observed experimentally or studied *in silico*. While they may be HPB-releasing [5,11,17], their thermal stability under hydrolysis conditions has not been confirmed. However, much of the evidence for POB adduct formation is based on mass spectra [6,9,37,41]. The mass spectra of POB and PTF adducts may be difficult to distinguish.

We have studied NNKFI as a potential intermediate in NNK metabolism and as a potential alkylating agent. We report the first investigation of NNKFI’s electronic structure, shape, vibrational properties and initial reactivity.

## 2. Methodology

### 2.1. Geometry optimizations

We obtained initial NNKFI coordinates from our previous work [38]. We then created five additional conformations by manually rotating the C3′–C5 torsion by 60°, 120°, 180°, 240° and 300° using UCSF Chimera version 1.11 [42]. These structures served as initial geometries for serial optimizations of increasing sophistication, beginning with HF [43,44]/3-21G* [45], moving through B3LYP [46–49]/6-31G* [50–52], and ending with B3LYP/6-311G** [53]. The reliability of the B3LYP functional has been documented previously [54]. The NWChem [55] computational suite was used to perform these calculations. Tight convergence criteria and frequency calculations were deployed. All reported geometries represent energy minima on NNKFI’s potential energy surface.

### 2.2. Reactivity descriptors

We assessed initial reactivity for NNKFI’s *cis* and *trans* conformers via a small slate of post-optimization analyses. Psi4 [56] and Multiwfn [57] were used to determine condensed Fukui functions and the condensed dual descriptor for each conformer. NBO 6.0 [58] was used to perform a natural population analysis for each conformer. Electrostatic potentials (ESPs) were visualized with UCSF Chimera v. 1.11. The 0.002 electron Bohr^−3^ isodensity surface was displayed. Graphical representations of energy-minimized structures were produced with ChimeraX [59,60].

### 2.3. Coupled cluster optimization and frequency calculation

To further confirm the validity of the B3LYP/6-311G** work, we performed coupled cluster geometry optimizations and frequency analyses using the GAMESS [61–63] computational chemistry suite. Our NWChem B3LYP/6-311G** results were utilized as initial geometries for the CCSD [64]/6-31G* optimizations. Graphical representations of selected vibrational modes were produced with wxMacMolPlt [65].

## 3. Results

Tables 1 and 2 report optimized energies and selected geometric parameters for NNKFI. Data are provided at the B3LYP/6-311G** and CCSD/6-31G* levels of theory. Rotation around the C3′–C5 bond produces two energetic minima. Dihedral ‘a’ was used to distinguish the conformers, a *trans* structure at approximately 180° and a *cis* structure at approximately 0°. Refer to the electronic supplementary material for graphical representations of the *cis* and *trans* structures.

**Table 1.** NNFKI structures optimized using the B3LYP/6-311G** and CCSD/6-31G* model chemistries. Letters a to e represent dihedral angles a: C2′−C3′−C5−O1, b: C3′−C5−C4−C3, c: C5−C4−C3−C2, d: C4−C3−C2−O1, e: C3−C2−O1−C5. 1 kcal = 4.14 kJ; Eh, hartree.

	energy		dihedral (°)				
	(kcal mol^−1^)	(Eh)	a	b	c	d	e
B3LYP/6-311G**							
*trans*	0	−478.81104	−179.8	−166.0	−24.1	24.6	−16.8
*cis*	0.54	−478.81018	0.3	−166.2	−23.8	24.4	−16.7
CCSD/6-31G*							
*trans*	0	−477.24043	−179.1	−165.9	−25.2	25.9	−17.6
*cis*	0.51	−477.23962	0.0	−165.6	−25.4	26.1	−17.8

**Table 2.** NNFKI backbone geometry reported at the B3LYP/6-311G** and CCSD/6-31G* levels of theory. Bond lengths are in ångstöm (Å) and bond angles are in degrees.

	B3LYP/6-311G**		CCSD/6-31G*	
bond	*trans*	*cis*	*trans*	*cis*
N1′–C2′	1.3222	1.3209	1.3316	1.3298
C2′–C3′	1.4149	1.4189	1.4111	1.4154
C3′–C4′	1.4151	1.4125	1.4143	1.4112
C4′–C5′	1.3788	1.3807	1.3830	1.3851
C5′–C6′	1.4021	1.4001	1.4049	1.4024
C6′–N1′	1.3404	1.3419	1.3444	1.3465
C3′–C5	1.4254	1.4257	1.4308	1.4310
C5–O1	1.2862	1.2860	1.2865	1.2864
C5–C4	1.4984	1.4982	1.5005	1.5002
C4–C3	1.5423	1.5423	1.5386	1.5386
C3–C2	1.5255	1.5259	1.5249	1.5248
C2–O1	1.4923	1.4912	1.4927	1.4921
N1′–C2′–C3′	123.25	123.09	123.23	123.04
C2′–C3′–C4′	118.25	118.20	118.89	118.85
C3′–C4′–C5′	118.29	118.46	117.89	118.09
C4′–C5′–C6′	118.53	118.42	118.57	118.45
C5′–C6′–N1′	124.06	124.08	124.35	124.35
C6′–N1′–C2′	117.61	117.74	117.06	117.22
C2′–C3′–C5	120.25	120.52	119.85	120.24
C4′–C3′–C5	121.49	121.28	121.26	120.91
C3′–C5–O1	119.31	119.53	119.07	119.36
C3′–C5–C4	128.55	128.34	128.68	128.42
O1–C5–C4	112.12	112.12	112.22	112.20
C5–C4–C3	102.84	102.89	102.51	102.49
C4–C3–C2	102.96	102.97	102.96	102.91
C3–C2–O1	103.72	103.75	103.56	103.52
C2–O1–C5	111.61	111.66	111.32	111.32

The B3LYP/6-311G** model predicts the more negative absolute energies, with the CCSD/6-31G* results approximately 1.6 Eh (hartree) less negative for each minimum. Relative energies (*trans* versus *cis*) are small in magnitude and show intermodel consistency, with the *trans* conformer about 0.5 kcal mol^−1^ (2.1 kJ mol^−1^) lower in energy than the *cis* conformer in both cases.

Dihedral angles in the furanium backbone show significant puckering, with atoms protruding above and below the plane defined by the pyridine ring. Dihedrals ‘b’, ‘c’, ‘d’ and ‘e’ are consistent for both conformers, varying by small fractions of a degree within a given model chemistry. Intermodel variations are larger for dihedrals ‘c’, ‘d’ and ‘e’, varying by as much as 1.7°.

Backbone bond lengths in the pyridine ring reveal a minor asymmetry between N1′–C2′ and C6′–N1′, with N1′–C2′ being slightly shorter. Likewise, C–C bond lengths in the pyridine ring show some variation, with the longest distances realized near C3′ and the shortest distances found near C5′. The C3′–C5 distance is approximately 1.43 Å in both conformers and both models. This value is slightly longer than any C–C separation found in the pyridine ring but is well below any seen in the furanium ring. The C5–O1 bond length is significantly shorter than the O1–C2 distance. C–C bonds in the furanium ring are also variable in length, reaching a maximum at C4–C3 and a minimum at C4–C5. Interconformer and intermodel bond length variations are minor for all reported values, differing by less than 0.01 Å.

Backbone bond angles in the pyridine ring show a minor asymmetry, with C5′–C6′–N1′ (124°) greater than N1′–C2′–C3′ (123°). Both angles are relatively large when compared with those found in the rest of the ring (118° or 119°). The C3′–C5 bond leans slightly toward N1′, with the C4′–C3′–C5 angle over 1° larger than the C2′–C3′–C5 value in both conformers and both models. Likewise, the carbonyl bond tilts away from the pyridine moiety, yielding a C3′–C5–C4 bond angle that is much larger than the C3′–C5–O1 angle in both conformers and both models. Some bond angles within the furanium ring show a similar asymmetry, with O1–C5–C4 (112°) and C3–C2–O1 (104°) differing notably. On the other hand, O1–C5–C4 and C5–O1–C2 are approximately equivalent at 112°. Likewise, C5–C4–C3 and C4–C3–C2 are both near 103°. Intermodel and interconformer angle variations are minor, with reported values differing by less than 1°.

We performed vibrational analyses on each reported geometry to confirm that they represent local minima on NNKFI’s potential energy surface. Table 3 reports selected vibrational frequencies for NNKFI’s *cis* and *trans* conformers. Data are given at the B3LYP/6-311G** and CCSD/6-31G* levels of theory. Unabridged vibrational analyses are found in the electronic supplementary material.

**Table 3.** Selected vibrational modes for NFKI. Data are reported for *cis* and *trans* isomers at the B3LYP/6-311G** and CCSD/6-31G* levels of theory. Reported modes involve bond stretching near C5 and O1.

	B3LYP/6-311G**		CCSD/6-31G*	
mode	*trans* (cm^–1^)	*cis* (cm^–1^)	*trans* (cm^–1^)	*cis* (cm^–1^)
17	852	855	883	885
45	1546	1547	1595	1596

Vibrational motion in NNKFI is somewhat difficult to characterize in canonical terms. The ion lacks symmetry and possesses 57 normal modes (excluding translation and rotation). The internal coordinates predicted for each conformer by each model chemistry are not entirely parallel, with mode ordering and atom displacement showing frequent intermodel variation and occasional interconformer variation. For visually analogous deformations, the B3LYP/6-311G** predicted frequencies are lower than their CCSD/6-31G* counterparts by approximately 20–50 cm^−1^. Frequency differences between conformers, on the other hand, are less than 20 cm^−1^.

We examined internal motion in NNKFI as a probe of bond strength, focusing on bond stretching near O1 and C5. We report vibrational modes that represent similar, though not identical, deformations in both conformers and at both levels of theory. Modes that include relevant atom displacements for only a subset of conformers or models are omitted. Two internal coordinates are listed in table 3. Mode 17 is an approximate O1–C2 stretching motion with predicted frequencies between 852 and 885 cm^−1^. Mode 45 is primarily a C5–O1 stretching coordinate with some C3′–C5 elongation. Predicted frequencies for this mode range from 1546 to 1596 cm^–1^. Graphical representations of the selected vibrations are given in the electronic supplementary material.

Table 4 reports condensed Fukui functions, dual descriptor and natural population analysis (NPA) charge for NNKFI calculated at the B3LYP/6-311G** level of theory. The condensed Fukui function for electrophilic attack exhibits significant interconformer variation peaking on atom C5′ in the *cis* structure and on atom N1′ in the *trans* structure. The condensed function for nucleophilic attack, on the other hand, shows relatively little variation between conformers and reaches its maximum value at C5. These results are mirrored in the dual descriptor entries with the most negative values found at C5′ in the *cis* conformer or N1′ in the *trans* conformer and the most positive values found at C5 in both structures. Since rotation around the C3′–C5 torsion is expected under ambient conditions, observed reactivity should be a mixture of these results.

**Table 4.** Atom condensed Fukui functions (ƒ^+^, ƒ^–^), dual descriptor (ƒ^+^– ƒ^–^), and NPA charge in units of *e* (1.602 x 10^−19^ C) for NNFKI. Entries are calculated at the B3LYP/6-311G** level of theory.

		*trans* NNKFI, B3LYP/6-311G**			*cis* NNKFI, B3LYP/6-311G**		
	NPA	ƒ ^–^	ƒ ^+^	ƒ ^+^ – ƒ ^–^	ƒ ^–^	ƒ ^+^	ƒ ^+^ – ƒ ^–^
N1′	−0.43	0.060	0.052	−0.008	0.292	0.052	−0.239
C2′	0.16	0.107	0.068	−0.038	0.063	0.070	0.007
C3′	−0.22	0.087	0.023	−0.065	0.050	0.023	−0.028
C4′	−0.07	0.052	0.068	0.016	0.060	0.067	0.006
C5′	−0.22	0.149	0.048	−0.101	0.068	0.047	−0.020
C6′	0.16	0.109	0.102	−0.007	0.059	0.102	0.043
C5	0.64	0.030	0.149	0.119	0.028	0.149	0.121
C4	−0.48	0.012	0.027	0.015	0.014	0.027	0.013
C3	−0.41	0.015	0.018	0.003	0.015	0.018	0.004
O1	−0.43	0.053	0.054	0.034	0.031	0.087	0.057
C2	−0.01	0.023	0.026	0.004	0.017	0.027	0.009

We report partial charges for the *trans* structure only. NPA charge shows little conformer dependence, varying by, at most, 0.02 units on any given atom. Significant positive character is seen on C5, C2′ and C6′ as well as on all hydrogen atoms (not shown). In contrast with the Lewis structure given in figure 1, negative character is realized on O1 and predicted charges reach their maximum value on C5.

**Figure 2.**
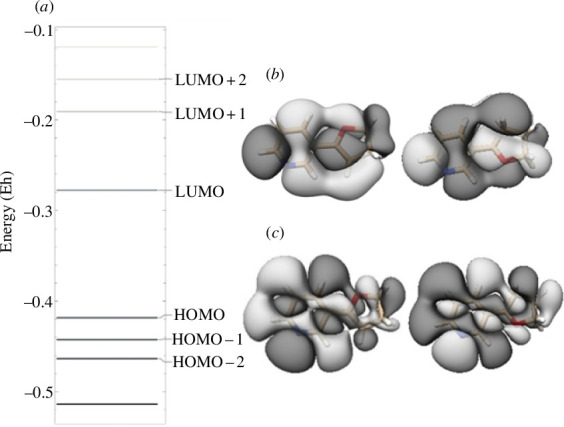
Energy diagram (*a*) for *trans* NNKFI and frontier orbitals for *trans* and *cis* NNKFI. Results are predicted at the B3LYP/6-311G** level of theory. Energy levels are shown on the left. The lowest unoccupied molecular orbital (LUMO) (*b*) is shown on the top right. The highest occupied molecular orbital (HOMO) (*c*) is shown on the bottom right.

Figure 2 shows a graphical representation of frontier orbitals for *trans* and *cis* NNKFI as well as a partial energy diagram for *trans* NNKFI, all predicted at the B3LYP/6-311G** level of theory. NNKFI is an electronic singlet. An attacking nucleophile is expected to donate electron density into NNKFI’s lowest unoccupied molecular orbital (LUMO). While this molecular orbital is quite delocalized, one of its lobes is approximately centred on C5.

**Figure 3.**
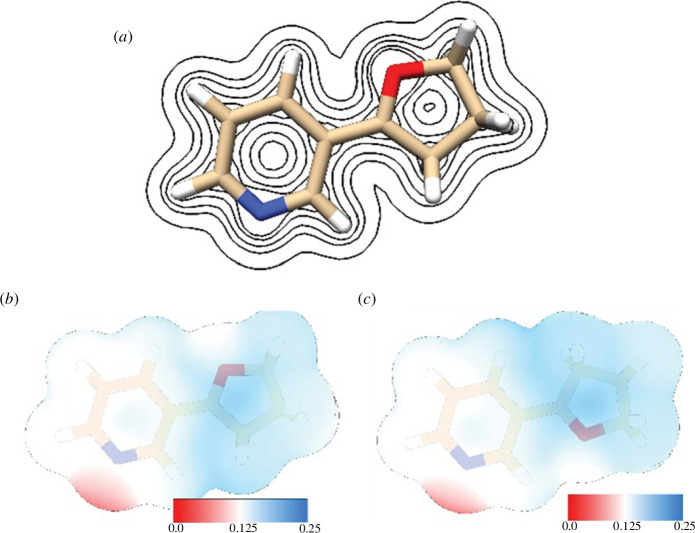
Electron density contours (*a*) and electrostatic potential (*b,c*) for NNKFI calculated at the B3LYP/6-311G** level of theory. Contour levels 0.01, 0.05, 0.1, 0.2, 0.3 in atomic units are shown (*a*). Electrostatic potential (atomic units) is visualized on NNKFI’s 0.002 electron Bohr^−3^ isodensity surface (*b,c*).

Figure 3 shows electron density contours (*trans* conformer) and ESPs (*trans* and *cis* conformers) for NNKFI. Results are calculated at the B3LYP/6-311G** level of theory. Regions of enhanced electron density extend outward from O1 and N1 but drop rapidly with distance from each respective nucleus. ESP on the 0.002 electron Bohr^−3^ isodensity surface is positive, with higher values found near the furanium ring but away from O1. Potential values on the pyridine ring system’s isodensity surface are relatively low, reaching a minimum near (but external to) N1. Potential values rotate with the furanium ring. This rotation does not appear to impact ESP on the pyridine moiety.

## 4. Discussion

NNKFI is expected to be susceptible to first-order nucleophilic substitution at C5 [5]. Resonance stabilization via carbocation formation at C5, C2′, C4′ or C6′ is the canonical chemical framework for understanding the reactivity of NNKFI. The ion’s π system is expected to extend into the furanium moiety. This extension should impact geometry and vibration in NNKFI. The magnitude of this effect may provide insight into the contribution of various structures to the resonance hybrid. Bond order and bond angles at or near the C5–O1 bond would likely be affected. We examine our findings and discuss their alignment with this framework.

Rotation around the C3′–C5 bond is expected to produce, at most, two minima. Our results show the expected *trans* and *cis* structures. The energy differences predicted for these conformers are consistent and small. Dihedral ‘a’ remains close to 0 or 180° for both models, indicating a planar relationship between C3′, C5 and O1 and supporting double-bond character in C3′–C5.

The values of dihedrals ‘b’ through ‘e’, on the other hand, imply rotation at C4, C3 and C2. This is indicative of ring puckering in the furanium moiety and suggests single bonds elsewhere in the furanium ring. Puckering has been experimentally observed in 2,3-dihydrofuran [66] and was anticipated in NNKFI. While the predicted dihedral angles are similar in both models and conformers, the CCSD/6-31G* geometry provides a somewhat less planar furanium ring than does the B3LYP/6-311G** structure. In addition, dihedrals ‘c’ though ‘e’ exhibit intermodel variation of up to 1.7°. The origin of these differences in shape and agreement is not clear.

Our geometric and vibrational predictions for NNKFI suggest a C5–O1 bond order that is significantly above unity. Given the lack of data on furanium ions, we have used neutral analogues for reference. Thus, oxonium ion formation and carbocation formation may both be used to explain some observed differences. Our predicted C5–O1 distance (1.29 Å), while longer than the carbonyl bond in formaldehyde (1.205 Å) [67], is significantly shorter than the C–O single bond found in 2,5-dihydrofuran (2,5-DHF, 1.4293 Å) [68]. The rest of our predicted bond lengths in the furanium backbone (1.49–1.54 Å) are modestly elongated analogues of the C–O and C–C single bonds experimentally measured in 2,5-DHF (1.4293 and 1.5013 Å, respectively) [68]. The elongation was consistent in both models and conformers.

Our reported harmonic vibrational frequencies reveal a marked difference in bond strength between O1–C2 and C5–O1. This points to a difference in bond order. Mode 45 (C5–O1 stretching) is found at 1546–1596 cm.^–1^ Mode 17 (O1–C2 stretching) is found at 852–885 cm^–1^. For comparison, the formaldehyde carbonyl stretch [69] occurs at 1746 cm^–1^ and the dimethyl ether symmetric C–O stretch [70] appears at 928 cm^–1^. Since neither of the predicted modes is a pure stretching displacement, we must use caution when interpreting the significance of these findings. Still, they are suggestive of a somewhat weakened carbonyl bond.

Predicted bond angles in the furanium ring align with an oxonium geometry. In particular, the similarity between the O1–C5–C4 and C5–O1–C2 angles (each 112°) as well as their contrast with the C5–C4–C3, C4–C3–C2 and C3–C2–O1 angles (103°–104°) are consistent with a double bond at C5–O1 and single bonds elsewhere in the furanium ring. Comparing our angle predictions with experimental values found in 2,5-DHF [68], we find that our C2–O1–C5 and C4–C5–O1 angles (112°) are similar to their C–C–C 2,5-DHF analogues (109.47°). Likewise, our C3–C2–O1 and C5–C4–C3 angles (104° and 103°, respectively) are close to their O–C–C analogues (105.41°). These results are also consistent with a C5–O1 double bond. However, our predicted C4–C3–C2 angle (103°) bears little resemblance to the experimentally determined C–O–C angle in 2,5-DHF (110.24°), where bond polarity is thought to produce repulsion between partial positive charges found on the carbon atoms. A similar effect may also enlarge the predicted C2–O1–C5 angle in NNKFI. Interconformer and intermodel variations are less than 1° and are not large enough to account for this difference.

Moving to the pyridine backbone, C–C bond lengths range from 1.38 to 1.42 Å, in agreement with the 1.39 Å experimental distance found in pyridine [71,72]. Distance trends, however, are different. In pyridine, C–C bond lengths show little variation, whereas our predicted C–C bond lengths increase with proximity to C3′. Predicted C–N bond lengths range from 1.32 to 1.34 Å, with shorter values found adjacent to C3′. These predictions are comparable to the 1.34 Å experimental value found in pyridine [71,72]. The furanium substituent is electron-withdrawing and the carbonyl bond is expected to participate in π resonance. These effects could alter backbone bond lengths in the pyridine ring. It is difficult to rationalize why they would impact C–C and C–N bonds differently, however. Similar bond length trends are observed in both conformers and models.

Predicted C–C–C angles in the pyridine moiety range from 118° to 119°, in agreement with the 118.4° to 118.5° experimental measurements found in pyridine [71,72]. Predicted C–C–N angles range from 123° to 124°, with smaller values found at C2′. This is in agreement with the 123.8° experimental value found in pyridine [71,72]. Our C–N–C angle predictions range from 117° to 118° and are slightly larger than the 116.9° experimental value found in pyridine [71,72].

We examined NNKFI’s ESP to gain insight into the physical interactions that precede electron exchange. Our predictions suggest that an approaching nucleophile would be directed towards the furanium moiety (where the ESP is highest) and away from N1′ [73]. The ESP on each isodensity surface reveals elevated potential in a broad, c-shaped region near, but not directly over, C5 for both conformers. Long-range electrostatic interactions between an approaching nucleophile and NNKFI are expected to aid in positioning the nucleophile in proximity to the C5 reactive site.

Our Fukui ƒ**^+^** predictions offer significant support for carbocation resonance in NNKFI. They show greater electrophilicity at C5 and C6′ than is seen at O1. Likewise, our NPA charge predictions reveal significant carbocation character at C5, C2′ and C6′. In aggregate, our Fukui ƒ **^+^**, NPA charge and ESP findings demonstrate significant delocalization of cationic charge in NNKFI and are consistent with reactivity towards approaching nucleophiles at C5.

Fukui ƒ**^+^** , ƒ^–^ and ƒ^+^ –ƒ^–^ values reveal conformer dependence in NNKFI. Atom O1 is slightly more susceptible to nucleophilic attack in the *cis* structure; N1′ is significantly more prone to electrophilic attack in the *cis* structure. Also, C2′, C5′ and C6′ are somewhat more subject to electrophilic attack in the *trans* conformer. This suggests that electrophiles would need to approach from above or below the pyridine ring to access the most reactive sites in the *trans* conformer.

In contrast, NPA charge and most Fukui ƒ**^+^** values show little interconformer variation. Thus, carbocation resonance does not offer a likely explanation for conformer-dependent initial reactivity towards nucleophiles. Likewise, NNKFI’s *cis* and *trans* LUMOs (figure 2) are quite similar in both appearance and energy. Therefore, an approaching nucleophile is expected to donate electron density into similar regions of either conformer. To explain interconformer variations in Fukui ƒ^–^ values, we must look elsewhere. The proximity of O1 and N1′ in the *cis* structure may allow donation of electron density from O1 to N1′, simultaneously boosting the electrophilicity of O1 and the nucleophilicity of N1′. Minor differences in the *cis* and *trans* highest occupied molecular orbitals (HOMOs) near O1 (figure 2) may enhance this effect. While both orbitals contain a planar node at O1, this node bisects the O1 lone pair in the *trans* structure.

## 5. Conclusions

We examined the electronic, structural, vibrational and chemical properties of NNKFI via *ab initio* calculations on its *cis* and *trans* conformers. Significant reactivity towards nucleophiles was found at C5. Geometric parameters, stretching modes and partial charges are consistent with a weakened carbonyl bond in an oxonium-like geometry. The impact of carbocation resonance on this species was analysed.

## Data Availability

The electronic supplementary material accompanying this article contains optimized *x*, *y*, *z* structures for all reported conformers and model chemistries [74]. Our calculations can be reproduced from these data and the open source software listed in the methods section.
